# Breakdown in the Organ Donation Process and Its Effect on Organ Availability

**DOI:** 10.1155/2015/831501

**Published:** 2015-04-09

**Authors:** Manik Razdan, Howard B. Degenholtz, Jeremy M. Kahn, Julia Driessen

**Affiliations:** ^1^Health Policy and Management, University of Pittsburgh Graduate School of Public Health, 130 DeSoto Street, A724B, Pittsburgh, PA 15261, USA; ^2^Health Policy and Management, University of Pittsburgh Graduate School of Public Health, 130 DeSoto Street, A616, Pittsburgh, PA 15261, USA; ^3^Critical Care, Medicine and Health Policy, University of Pittsburgh School of Medicine, 3550 Terrace Street, Pittsburgh, PA 15261, USA; ^4^Health Policy and Management, University of Pittsburgh Graduate School of Public Health, 130 DeSoto Street, Pittsburgh, PA 15261, USA

## Abstract

*Background*. This study examines the effect of breakdown in the organ donation process on the availability of transplantable organs. A process breakdown is defined as a deviation from the organ donation protocol that may jeopardize organ recovery. *Methods*. A retrospective analysis of donation-eligible decedents was conducted using data from an independent organ procurement organization. Adjusted effect of process breakdown on organs transplanted from an eligible decedent was examined using multivariable zero-inflated Poisson regression. *Results*. An eligible decedent is four times more likely to become an organ donor when there is no process breakdown (adjusted OR: 4.01; 95% CI: 1.6838, 9.6414; *P* < 0.01) even after controlling for the decedent's age, gender, race, and whether or not a decedent had joined the state donor registry. However once the eligible decedent becomes a donor, whether or not there was a process breakdown does not affect the number of transplantable organs yielded. Overall, for every process breakdown occurring in the care of an eligible decedent, one less organ is available for transplant. Decedent's age is a strong predictor of likelihood of donation and the number of organs transplanted from a donor. *Conclusion*. Eliminating breakdowns in the donation process can potentially increase the number of organs available for transplant but some organs will still be lost.

## 1. Introduction

The success of organ transplants in treating end-stage organ failure has led to an unprecedented demand for transplantable organs that unfortunately remain in short supply. As a result, relatively few organs are transplanted compared to the number of people with end-stage disease. In 2012, more than 116,000 patients were on the United States transplant waiting list but only about 28,000 transplants were performed [1]. Consequently 6,508 patients died while waiting for a life-saving organ [1]. Increasing the availability of transplantable organs is therefore critical in preventing deaths from end-stage organ failure. Thus, it is an urgent and an ongoing concern that the opportunity for organ donation is preserved when caring for critically ill patients.

Estimates of organ donation potential indicate that there is a sizeable potential donor pool that the organ donation community is yet to completely realize. In the US (1997–1999), there were estimated 40,610 brain-dead potential organ donors but only 17,127 actual donors [2]. Poor relationship between key organizations including organ procurement organizations (OPOs) and hospitals in their donation service area (DSA) is one of the several reasons for this tragic loss of donors [3]. In 2001, the Organ Donation Breakthrough Collaborative was established to, among other reasons, encourage collaboration between the OPOs and donor and transplant hospitals [4]. Although considerable increase in the number of organs available for transplantation has been realized since the collaborative began [5–9], acute shortage of organs continues to be a major challenge for the transplant community.

In recent years critical pathways for organ donation have been developed to preserve the opportunity for donation. Critical pathways, also called “clinical pathways” or “care maps,” help standardize medical care, reduce variability, and improve outcomes of medical procedures [10]. In the US, standardized protocols or best practices (see* Best Practices in Donation Process* below) in organ donation have been jointly developed by the OPOs and their partnering hospitals. OPOs invest considerable resources in training hospital staff in following these best practices, about their roles in the organ donation process, and on how to eliminate errors in patient care that may jeopardize the potential for donation. Although there is a committed and a quality-oriented culture, competing priorities in the hospital and the inherent complexity of critical care can sometimes lead to deviations from the best practices. OPOs identify these deviations as “process breakdowns.” While several studies have identified a relationship between process breakdowns and conversion rate (the actual number of organ donors divided by the number of eligible deaths) [11–15], it is unclear how process breakdowns affect the supply of viable organs.

*Best Practices in Donation Process*. Consider the following:

 prompt identification of imminent death patients,
 timely notification to the OPO (within 1 hr. of identifying imminent death patient),
 notifying the OPO about every death,
 early and aggressive potential donor management,
 timely and designated family approach (optimal request for organ donation).

Using patient-level data from an OPO, we examined the effect of process breakdowns on the supply of transplantable organs. Specifically, we examined the effect of process breakdowns on two stages in the organ donation process: (1) eligible decedent's family agrees to donation and (2) organs from the donor are transplanted. We note that, for reporting purposes, the Centers for Medicare and Medicaid Services (CMS) identifies an organ donor as an eligible decedent whose family has authorized recovery of organs for transplantation. Indeed all organ donors in our dataset had some organs recovered for transplantation irrespective of whether they were actually transplanted. We use the term “donor” in similar sense in this paper meaning that an eligible decedent may become a donor and still not yield any transplantable organs.

## 2. Materials and Methods

A retrospective analysis of decedents was conducted using data from the Center for Organ Recovery and Education (CORE), an independent OPO that serves western Pennsylvania and most of West Virginia. All deaths from January 1st 2010 through December 31st 2012 were considered for the analysis. All data manipulation and analytic procedures were performed using Stata SE 13.0 (StataCorp, College Station, Texas).

### 2.1. Data

Decedent records were extracted from CORE's data system for analyses. Like hospitals, CORE uses a proprietary electronic medical record system to aid its staff in documenting real-time patient-related information. The information is jointly entered into the system by the donor referral coordinator and the organ procurement coordinator. Decedents who had died a CMS-defined “eligible death” were included in the analyses. CMS defines eligible death as a brain-dead individual up to age 70 who does not exhibit any of the exclusionary conditions (active infections, cancers, etc.) listed in CMS Conditions for Coverage for the OPOs (42 CFR §486.302). CMS-defined eligible deaths become standard criteria donors. A justification for the exclusion of nonstandard criteria donors is available in optional supplemental materials in the Supplementary Material available online at http://dx.doi.org/10.1155/2015/831501.

### 2.2. Variables

For each eligible decedent, information was retrieved on age, gender, race, hospital where death occurred, organ donor status, whether the decedent had joined the state donor registry (registered decedent), organs transplanted, and whether there was a process breakdown. The outcome variable of interest is the number of organs transplanted from an eligible decedent. The predictor variable is whether there was a process breakdown in the care of an eligible decedent.  Table 1 presents a list of process breakdowns that CORE's personnel identify, document, and resolve on a day-to-day basis. For missed referrals, untimely referrals, and suboptimal request for donation, CORE assesses whether or not referral and request requirements specified in CMS Conditions of Participation for Hospitals (42 CFR §482.45) were met. Deescalation of care and early extubation are documented as process breakdowns if either happens before a request for donation is made to the family.

### 2.3. Descriptive Analyses

First, summary statistics were computed for age, race, gender, organ donors, organs transplanted, and process breakdowns. Next, donors were compared with nondonors, and those who had joined the state donor registry were compared with those who had not, for age using Wilcoxon Mann-Whitney rank-sum test and for race and gender using the Chi-squared test. The unadjusted effect of process breakdown on the probability of becoming a donor was examined using Chi-squared test and on the number of organs transplanted using Wilcoxon Mann-Whitney rank-sum test.

### 2.4. Regression Framework

We used zero-inflated Poisson regression to examine the effect of process breakdown on organs transplanted. The organ donation process is a two-stage process. In the first stage, an eligible decedent becomes an organ donor after the decedent's family authorizes donation. Where the family refuses donation, there is no possibility of recovering any organs from the decedent resulting in “structural zeroes” in the outcome variable. In the second stage, organs from a donor are offered for transplantation. Depending on several factors including viability of the organs offered, the potential recipient's health, and so forth, organs are either rejected or accepted for transplant. When no organs are transplanted from a donor, “sampling zeroes” arise. The outcome variable therefore represents a mix of structural and sampling zeroes that can be appropriately modelled using a zero-inflated regression framework. When organs are accepted, the outcome variable assumes discrete positive values and organs transplanted per donor assume a Poisson distribution (discrete nonnegative values with the mean of the distribution equal to its variance).

The choice of covariates to be included in the model was informed by theory, results of the bivariate analysis, and* a priori* assumption and confirmed using model fit statistics. For modelling structural zeroes (family refusal to donate), the variable age, which is a known determinant of the likelihood of becoming an organ donor, was included as a covariate [16, 17]. Other covariates included were whether or not a decedent had joined the state donor registry and being a Caucasian since these covariates were significantly different between donors and nondonors in the bivariate analysis. For modelling the organs transplanted per donor, only age was included as a covariate based on* a priori *belief that older decedents will yield less viable organs. Intrahospital correlation between patients and their families was treated as nuisance and accounted for by computing clustered standard errors.

The appropriateness of covariate selection was examined by computing several fit statistics including Cragg and Uhler's Pseudo-R-squared, McFadden's Adjusted R-squared, AIC, BIC, and model Deviance. To assess the overall goodness-of-fit, the sensitivity of the regression results to alternative regression frameworks (zero-inflated negative binomial and two-part model) was examined.

## 3. Results

### 3.1. Descriptive Statistics

**Figure 1:**
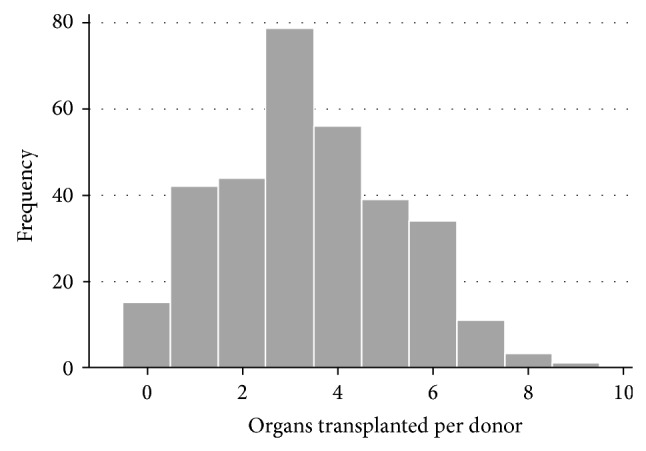
Donors as a subset of eligible deaths and all decedents.

Out of the 84,817 deaths reported to CORE between January 1st, 2010, and December 31st, 2012, there were only 424 eligible deaths of which 324 (76.4%) went on to become organ donors. As a result, 1,100 organs were transplanted in the three-year period. Among the 324 donors, 15 donors did not yield any transplantable organ (sampling zeroes). The mean number of organs transplanted per donor was 3.40 and the variance was 3.41 suggesting that Poisson regression (in the second part of the model) is appropriate.  Figure 1 presents the distribution of organs transplanted from donors. Summary statistics for age, gender, race, registered decedent status, and process breakdowns are presented in Table 2. Comparison of donors with nondonors and registered decedents with nonregistered decedents is presented in Tables 3 and 4.

#### 3.1.1. Process Breakdown and the Probability of Becoming an Organ Donor

Overall, 76 percent of eligible decedents became organ donors. This proportion was slightly higher (79 percent) when there was no process breakdown but dropped to 36 percent when a process breakdown had occurred. There were 25 eligible decedents who had experienced a process breakdown in their care. Of these, only 9 decedents went on to become organ donors. Chi-squared test of conversion rate by process breakdown indicates a significant association between the two. An eligible decedent was 6.7 times more likely to become an organ donor if there was no process breakdown in the care of the patient (unadjusted OR: 6.67; 95% CI: 2.85, 15.62; *P* < 0.001).

#### 3.1.2. Process Breakdown and Organs Transplanted

Overall, a mean of 2.59 organs were transplanted from an eligible decedent (including eligible decedents who did not become organ donors), 52 percent of whom yielded at least 3 transplantable organs. In the presence of a process breakdown, the mean number of organs transplanted from an eligible decedent dropped to 1.16 with only a quarter of eligible decedents yielding 3 transplantable organs. When there was no process breakdown, 2.68 organs were transplanted per eligible decedent on average. Bivariate analysis using Wilcoxon Mann-Whitney rank-sum test indicates that an eligible decedent is expected to yield significantly greater number of organs when there is no process breakdown (*P* < 0.001).

### 3.2. Zero-Inflated Poisson Regression

Holding other variables constant, the likelihood that an eligible decedent will become an organ donor is four times higher when there is no process breakdown (adjusted OR: 4.01; 95% CI: 1.6838, 9.6414; *P* < 0.01). However once a decedent becomes a donor, whether there was a process breakdown does not affect the number of transplantable organs yielded by the donor. Regression results are presented in Table 5. In spite of effect-loss at the second stage in the organ donation process, process breakdowns exert a strong detrimental effect on organs transplanted. For every process breakdown an eligible decedent yields around one less organ (*dy*/*dx*: −1.05; 95% CI: −2.0307, −0.0706; *P* < 0.05). Age has a small but strong effect on organs transplanted per eligible decedent. Joining the registry had a significant positive impact on the odds of becoming a donor. Decedent's race was not significant. Incremental and marginal effects are presented in Table 6.

Fit statistics on various nested models are presented in Table 7. Results from sensitivity analyses using different regression frameworks are presented in Table 8. The coefficient estimates are almost identical for the predictor variable and other covariates (except joining the registry) under the zero-inflated Poisson, zero-inflated negative binomial, and two-part model.

## 4. Discussion

Evidence from published evaluations of quality improvement initiatives undertaken by several OPOs suggests that there is an inverse association between process breakdowns and donation rate [11–15]. Burris and Jacobs found that a mandatory twenty-minute training for staff in nursing, patient and family services and pastoral care, and employing compliance monitoring tools resulted in an increase in the referral rate from 54 percent to 98 percent over a 10-month period. During the same time, donation rate for all decedents between 6 months old and 76 years old increased from 1.6 percent to 3.1 percent [11].

Sade et al. examined the effect of specialization within the procurement process on the consent rates in an OPO's DSA [12]. Between 1997 and 2001, after clinical services liaisons were recruited to educate the hospital staff about the donation process and review medical records for appropriateness of referrals, the number of referrals for all deaths increased by 49 percent. In addition, specialist family support counselors approached the family with the request for donation resulting in 90 percent increase in the consent rate. During the same period, the donation rate per million population also increased by 83 percent [12].

Similar association between timely referrals and organ donation rates was suggested by Koh et al. in their assessment of the Massachusetts Organ Donation Initiative [13]. The program was a data-driven quality improvement program that involved coordination between the Massachusetts Department of Health, the regional OPO, and the transplant centers [13]. They found that delayed referral and suboptimal request for donation accounted for most lost donation opportunities. The hospital liaisons implemented changes that increased referral rates from 83 percent to 94 percent. As a result the authorization rate increased from 60 percent to 67 percent, and conversion rate increased from 44 percent to 60 percent [13].

Franklin et al. found that systematic protocol driven changes to the organ donation process gradually improved organ donation rates across the OPOs DSA [14]. Changes relevant to best practices in organ donation included decoupling of the request process, family approached by clinical coordinator, and family support liaisons. Between 1993 and 2008, the conversion rate increased from 42 percent to 72 percent [14].

In one study however, researchers did not find an association between timely referral/appropriate family approach and the conversion rate. Although implementing evidence-based best practices resulted in significant improvement in conversion rate (from 50 percent in 2004 to 80 percent in 2005), the referral rate, timely notification rate, and the appropriate requester rate did not show significant improvement [15]. However, since that study only included 32 eligible decedents in the preimplementation group and 30 eligible decedents in the postimplementation group, it probably lacked sufficient power to detect the small improvements.

Our study differs from the existing literature in two important ways. First, the existing literature is based on before-after comparison of donation rates at the level of the hospital or the DSA. We reexamined the relationship between process breakdowns and donation rates by using DSA-wide decedent level data to adjust for other patient factors that are known to affect organ donation rates. Second, we also examined the effect of process breakdowns on organs transplanted per eligible decedent, which has not been previously studied.

Our analysis indicates that process breakdowns significantly reduce the likelihood of organ donation but have no effect on the number of organs transplanted. The loss of effect in the second stage is best explained by how different process breakdowns are distributed. While 25 eligible decedents had experienced a process breakdown, only 9 of those decedents went on to become organ donors. Eight of these nine organ donors had experienced either a delayed referral or a suboptimal request for donation. Since delayed referrals and suboptimal requests, in theory, do not affect organ health or function, their insignificance in the second stage of the regression model was somewhat expected. Nevertheless we wanted to investigate this relationship to determine if there were other mediating factors through which these process breakdowns could exert their influence. For instance, delayed referral of an imminent death can delay identification of a potential donor, brain death testing, and donor management, all of which will adversely affect organ function. Similarly, suboptimal request for donation may either result in family's refusal to donate or more time spent on obtaining authorization resulting in a delay in organ retrieval. Even in optimally managed brain-dead donors, delay in retrieving organs can compromise organ quality. Although process breakdowns do not affect the number of organs transplanted once the family has authorized donation, the overall effect of process breakdowns is still significant owing to their strong effect on the likelihood of becoming a donor. This observation is consistent with the results of bivariate analysis where the effect of process breakdown on the number of organs transplanted from an eligible decedent was significant.

Age at death exerted significant influence on the likelihood of becoming a donor as well as organs transplanted per donor. Our results indicate that older decedents are less likely to become donors than younger decedents. For every one year increase in age, the likelihood of organ donation steadily declined by 4 percent. Since all decedents in our dataset were, by definition, eligible to become donors, the effect of age on becoming a donor is probably mediated through the fact that families of younger patients tend to authorize donation more often than families of older patients [16, 17]. This effect can be explained in part by a higher proportion of traumatic accidental deaths in the younger patients compared to older patients who die more often from a medical cause. In CORE's DSA between 2010 and 2012, the median age of donors who died from traumatic head injury was 28 years. In contrast, the median age of donors who died from a cerebrovascular accident or stroke was 59 years. Families of decedents who are brain dead from a medical cause (e.g., stroke) have greater difficulty in understanding the concept of brain death and are more resistant to donation [17]. In addition, older decedents are likely in poorer health, also potentially leading to fewer organs transplanted per donor.

Our results indicate that race and gender do not predict the likelihood of organ donation. Previous evidence suggests that women are more willing to donate their organs and discuss their willingness to donate with their families [18]. Since having knowledge of the deceased's wishes facilitates authorization for donation [16, 19, 20], we had expected female eligible decedents to have higher adjusted odds of organ donation. Contrary to previous studies [17, 21, 22], we did not find race to be a significant predictor either. While donors and nondonors in our data differed significantly by race (Caucasian), these differences disappeared when designated donor status was included in the regression model. Since Caucasians are more likely to have joined the state donor registry, the effect of being a Caucasian is mediated through the donor designation status, a significant covariate in the regression model (*P* = 0.052).

Although process breakdowns reduce the likelihood of donation, a significant observation is that even if process breakdowns are completely eliminated, some organ donors will still be lost owing to other factors that make families averse to donation. These factors which are largely outside the control of the OPOs and the hospitals include patient and family demographics [19, 20], family's religious beliefs [20], fear of mutilation and high value placed on bodily integrity [20, 23], concern about use of organs and distrust of medical community [20], family not knowing decedent's wishes [16, 19, 20], poor understanding of brain death [20, 24], bereaved family's emotional state [20], and family disagreement regarding donation decision [25]. Understanding of brain death is especially important because higher authorization rates cannot be achieved unless the relatives of the decedent understand that there is no hope of recovery [26]. In contrast, when decedents have their name on the state donor registry, family's decision about donation becomes irrelevant owing to the legal guarantee afforded under the Uniform Anatomical Gift Act of 1987. It is therefore not surprising that 100% of eligible decedents who had joined the state donor registry became donors.

Our first choice of regression framework for modelling the two-stage organ donation process was a two-part hurdle model where, in the first part, we would model the likelihood that an eligible decedent becomes a donor using logistic regression, and in the second part, we would use Poisson or negative binomial regression to model the organs transplanted per donor. Two-part models are commonly used in modelling healthcare costs where majority of individuals, who do not utilize medical care in a given year, have zero healthcare costs. The zeroes observed in these data are purely structural and the two-part model evaluates the zero observations separate from nonzero observations. However, since the zeroes observed in our data are a mix of structural and sampling zeroes, a hurdle model would misclassify some of the donors who did not yield transplantable organs as nondonors. For our data, a zero-inflated model is more appropriate since it models only those zeroes separately that are in excess of what would be expected based on the remaining nonzero observations; that is, some sampling zeroes are expected. In essence, a zero-inflated model is a two-part model where the assumption that all zeroes are structural is relaxed.

Hospital-level differences were not controlled by inclusion as a covariate or using fixed effects. There are two possible mechanisms through which hospital-level differences might affect organ donation rates. First, factors such as ownership, mission (profit versus nonprofit), level of trauma services, and having a transplant program are known to be associated with a wide range of indicators of organ procurement performance [27]. However these factors are unlikely to affect our estimates since their effect on donation rates is mediated through process breakdown. OPOs maintain in-house coordinators in underperforming hospitals with large donation potential for the same reason, that is, to reduce process breakdowns so that donation rates can be improved. Model fit statistics suggest that our choice of covariates in the model is reasonable. In addition, sensitivity analyses with alternative regression frameworks suggest that our model is robust to alternative assumptions and therefore has a reasonably good fit. We did not use split-sample cross-validation to assess overall goodness-of-fit owing to a small sample size and rarity of process breakdowns.

### 4.1. Limitations

This study was based on data from one OPO that serves western Pennsylvania and West Virginia. The results of our study may have limited generalizability outside this geographic region. In spite of efforts at dissemination and adoption of emerging state-of-the-art practices, there are wide variations in organ donor practices across different DSAs, including the way process breakdowns are defined and recorded.

In CORE's DSA, process breakdowns are documented and recorded by the procurement coordinators themselves. This poses two potential issues. First, there is a potential for measurement error if different staff interprets the categories differently or fail to record situations in order to protect themselves or to avoid conflict with the hospital staff. However, we believe that the likelihood of wide variation in classification of process breakdowns is low, due to the close teamwork among CORE's professional services liaison staff, use of a standardized electronic data system, and the fact that the data are auditable by CMS. The second issue is the potential risk of recall bias if the procurement coordinator's documentation of a process breakdown is correlated with whether or not an eligible death becomes a donor. To assess if this bias was affecting our analysis, we compared the eligible decedents with the noneligible decedents for the fraction of process breakdowns. Three percent of all noneligible deaths had a documented process breakdown while this fraction was slightly higher (4.1%) for eligible deaths (*P* > 0.05) suggesting that there is a great degree of independence between documenting the process breakdown and the outcome of a particular case.

## 5. Conclusion

The principal finding of this study is that process breakdowns have a strong adverse effect on the likelihood of organ donation but do not affect the organ yield once an eligible decedent becomes an organ donor. Nevertheless, process breakdowns exert a strong overall effect on organ availability. Our results suggest that, for every process breakdown occurring in an eligible decedent, one less organ is available for transplant. Accordingly, we estimate that 25 organs were lost to process breakdowns over a three-year period. While the adverse effect of process breakdown on donation rates has been previously studied, this is the first study to quantify this effect in terms of the number of organs lost.

Another finding of significance is that the donation rate was 79 percent in the absence of process breakdowns. This indicates a diminishing marginal return on eliminating process breakdowns. That is, for every additional dollar that is invested in reducing process breakdowns, the effect is progressively decreasing. On the other hand, when an eligible decedent had joined the state donor registry prior to death, the donation rate equaled 100 percent. These findings have important implications for resource allocation decisions that OPOs have to make, for example, spending on hospital education to reduce process breakdowns versus spending on educating and encouraging the public to join the state donor registry. Further research in this area will help inform OPOs' resource allocation decisions.

Needless to say, resource allocation decisions mentioned above are valid in the broader US organ donation legislative framework where OPOs are under legal obligation to recover organs from designated donors. In countries where the statement of intent is not legally binding, promoting donor registries might offer no marginal benefit over reducing process breakdowns.

## Supplementary Material

A justification for the exclusion of nonstandard criteria donors.

## Figures and Tables

**Table 1:** Process breakdowns and determination criteria.

Name	Description	Determination
Missed referral	The OPO was never notified about the deceased.	Unaccounted patient deaths are found during medical/death record review by the hospital development staff.

Untimely referral	The OPO was not notified about the imminent death within 1 hour of such determination, or if the patient has died, within one hour of death.	The donor referral coordinator who receives the call from the hospital verifies the time of imminent death determination, or if the patient has died, the time of patient's death. These times are then compared with the time when the hospital notifies the OPO.

Suboptimal request for donation	Either the timing of the request is poor or the person requesting donation is not a trained requestor. Poor timing of the request includes discussing donation either before or soon after the family is informed about patient's death.	These process breakdowns are either self-reported by the hospital staff (e.g., “Dr. Doug mentioned organ donation to the family”) or by the family to the procurement coordinator (“We have been asked about donation and we don't want to do it”).

Deescalation of care	The referral is made timely but hemodynamic stability is not maintained and life-saving measures are discontinued. Only comfort measures are provided.	While assessing patient's medical record, the procurement coordinator finds that the patient is on “comfort only” measure.

Early extubation	Initial referral is made on time but the patient is withdrawn from the ventilator before the family is offered the opportunity to donate.	The procurement coordinator records that the patient was removed from the ventilator and passed away before request for organ donation is made to the family.

**Table 2:** Descriptive statistics for eligible deaths.

Median age	**37** years
Females	40%
Joining the state donor registry	32%
Race	
Caucasians	87%
African-Americans	11%
Process breakdowns (*N*)	25
Suboptimal request	17
Untimely referral	5
Deescalation of care/early extubation	1
Unidentified	2

**Table 3:** Difference between donors and nondonors.

	Donors	Nondonors	*P* value
Median age	35 years	47 years	0.000^a^
Females (%)	42%	33%	0.124^b^
Caucasians	89%	78%	0.004^b^

^a^Wilcoxon Mann-Whitney rank-sum test (test of medians).

^b^Chi-squared test.

**Table 4:** Difference between registered and nonregistered donors.

	Registered donors	Nonregistered donors	*P* value
Median age	39 years	35 years	0.058^a^
Females	47%	37%	0.040^b^
Caucasians	95%	83%	0.001^b^

^a^Wilcoxon Mann-Whitney rank-sum test (test of medians).

^b^Chi-squared test.

**Table 5:** Results from zero-inflated Poisson regression.

The likelihood of becoming an organ donor				
	Log odds	Clustered Std. Err.	*P* value	95% C.I.
Process breakdown	−1.39	0.4452	0.002	−2.2663, −0.5211
Age	−0.04	0.0101	0.000	−0.0597, 0.0202
Joined the registry	3.94	1.7474	0.024	−0.5172, 7.3667
Caucasian	0.51	0.357	0.129	−0.1489, 1.1732

Organs transplanted per donor				
	Log counts	Clustered Std. Err.	*P* value	95% C.I.

Process breakdown	−0.16	0.1742	0.355	0.5027, 0.1802
Age	−0.01	0.0027	0.000	−0.1832, −0.0076

*N* = 424.

**Table 6:** Incremental and marginal effects.

	ExpOrgTx^a^	Std. Err.	*P* value	95% C.I.
Process Breakdown	−1.05	0.5000	0.036	−2.0307, −0.0706
Age	−0.05	0.0085	0.000	−0.0684, −0.0351
Joined the registry	1.79	0.7243	0.014	0.3675, 3.2067
Caucasian	0.23	0.1511	0.124	−0.0640, 0.5283

*N* = 424.

^a^Expected number of organs transplanted from each eligible decedent.

**Table 7:** Statistics for model fit.

Excluded variable(s)	Cragg & Uhler's pseudo *R* ^2^	McFadden's Adj. *R* ^2^	AIC	BIC	Deviance
None (saturated model)	**0.269**	**0.065**	**3.708**	**−948.87**	**1548.69**
Age	0.156	0.032	3.840	−901.52	1608.15
Joined registry	0.171	0.036	3.826	−903.06	1600.55
Caucasian	0.266	0.065	3.709	−952.88	1550.74
Female^∗^	0.270	0.068	3.694	−966.37	1550.32
Female, Caucasian	0.270	0.068	3.694	−970.38	1552.36

^∗^Being a female was excluded from the final model.

**Table 8:** Sensitivity analysis-regression framework.

	ZIP		ZINB		TPM	
Likelihood of becoming a donor						
	Log odds	*P* value	Log odds	*P* value	Log odds	*P* value
PBD	−1.39	0.002	−1.39	0.002	−1.38	0.002
Age	−0.04	0.000	−0.04	0.000	−0.04	0.000
Joined registry	3.94	0.024	3.94	0.024	2.40	0.000
Caucasian	0.51	0.129	0.51	0.129	0.51	0.151

	ZIP		ZINB		TPM	
Organs transplanted per donor						
	Log count	*P* value	Log count	*P* value	Log count	*P* value

PBD	−0.16	0.355	−0.16	0.355	−0.14	0.337
Age	−0.11	0.000	−0.11	0.000	−0.01	0.000

ZIP: zero-inflated Poisson regression.

ZINB: zero-inflated negative binomial regression.

TPM: two-part model.

## Abbreviations

OPO: Organ procurement organization
DSA: Donation service area
CORE: Center for Organ Recovery and Education
CMS: Centers for Medicare and Medicaid Services.
