# Supplementary material for: Breakdown in the Organ Donation Process and Its Effect on Organ Availability
Source: J Transplant. 2015 Apr 9;2015:831501. doi: 10.1155/2015/831501 (PMC4407530; doi:10.1155/2015/831501)

**Justification for excluding non-standard criteria donors**

To be reimbursed through the Medicare and Medicaid programs, organ procurement organizations must meet the requirements established in the Centers for Medicare and Medicaid Services (CMS) Conditions for Coverage for OPOs (42 CFR §486.318). One of the Conditions for Coverage is that OPOs must have a donation rate that is not significantly lower than the national average for all OPOs. The donation rate, also called the conversion rate, is defined as the fraction of eligible deaths that become organ donors. CMS defines an eligible death as a patient 70 years old or younger, who is legally declared brain dead, and who does not exhibit any of the exclusionary conditions listed in CMS Conditions for Coverage for OPOs (42 CFR §486.302). Donation rate allows CMS to assess how well an OPO has performed when compared to other OPOs. An organ donor who meets the eligible death criteria is called a standard criteria donor (42 CFR §486). The way eligible death and standard criteria donor (SCD) are defined under 42 CMS §486, eligible deaths can only result in standard criteria donors and standard criteria donors are always a subset of eligible deaths.

Individuals who do not die an eligible death can also become organ donors. These donors are classified as either donors after cardiac death or expanded criteria donors. Donors after cardiac death are declared dead using the circulatory determination of death criteria set forth in the Uniform Determination of Death Act. Expanded criteria donors are brain dead donors that do not fit the standard criteria donor profile. The Venn diagram in Figure S1 represents the relationship between all decedents, brain-dead decedents, eligible decedents, and the three types of donors. Relative sizes of the different pools are correctly depicted but are not to scale.

CMS does not define eligible deaths for donors after cardiac death and expanded criteria donors. For the lack of an objective definition, these eligible deaths are neither observed in the real world nor in our data. Eligible deaths observed in our data can only result in standard criteria donors. As a result, donors after cardiac death and expanded criteria donors lack a superset of eligible deaths. Since only standard criteria donors have an eligible death superset, we will restrict our analysis to estimating the effect of process breakdowns on the availability of organs from standard criteria donors. Including donors after cardiac death and expanded criteria donors in the regression model will produce biased estimates of the effect.

Supplemental Figure S1: Donors as a subset of eligible deaths and all decedents


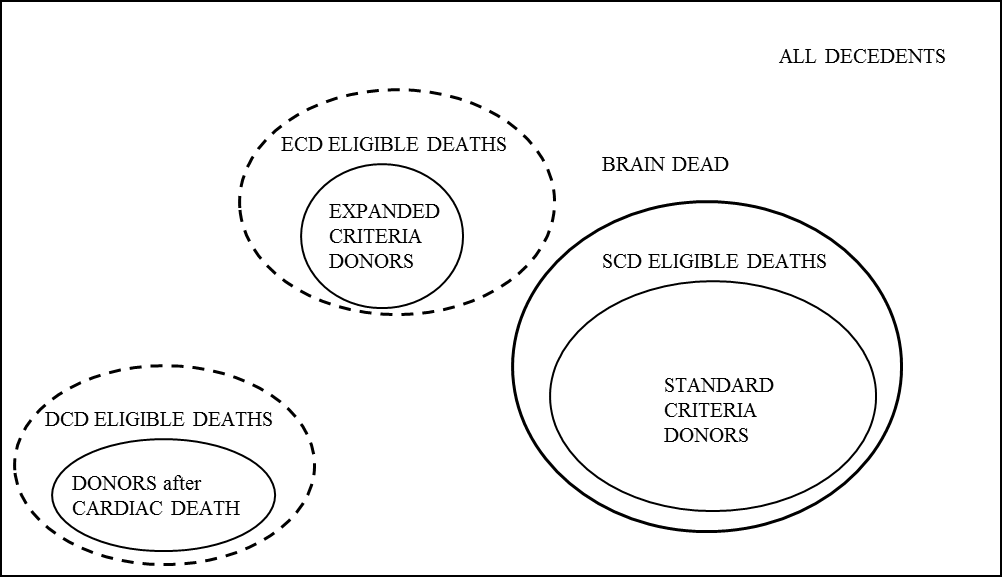

Supplement: Supplementary file 1 — A justification for the exclusion of nonstandard criteria donors. [file 831501.f1.docx]
